# Toxic Effect of Cigarette Smoke on Brainstem Nicotinic Receptor Expression: Primary Cause of Sudden Unexplained Perinatal Death

**DOI:** 10.3390/toxics6040063

**Published:** 2018-10-18

**Authors:** Anna Maria Lavezzi

**Affiliations:** “Lino Rossi” Research Center for the Study and Prevention of Unexpected Perinatal Death and SIDS, Department of Biomedical, Surgical and Dental Sciences, University of Milan, 20122 Milan, Italy; anna.lavezzi@unimi.it; Tel.: +39-025-032-0821

**Keywords:** nicotine, nicotinic acetylcholine receptors, immunohistochemistry, sudden fetal death, sudden neonatal death, brainstem, Kölliker-Fuse nucleus

## Abstract

Among the neurotoxicants contained in tobacco smoke, if absorbed during pregnancy, nicotine significantly affects α7-nicotinic acetylcholine receptors, which play essential roles in the development of the brainstem regions receiving cholinergic projections in perinatal life. Immunohistochemical procedures for analysing formalin-fixed and paraffin-embedded brainstem samples from 68 fetuses and early newborns, with smoking and non-smoking mothers, who died of known and unknown causes, were carried out in order to determine if nicotine had activated the α7-nicotinic acetylcholine receptors. High α7-nicotinic acetylcholine receptor expression levels were only observed in the victims with smoking mothers. Frequently, these findings were associated with the hypoplasia of the brainstem structures controlling vital functions. The results of this study indicate that the exposition to nicotine in pregnancy exerts a strong direct effect on α7-nicotinic acetylcholine receptor activity especially in perinatal life and may be one of the primary risk factors leading to the sudden unexplained death of fetuses and newborns.

## 1. Introduction

To date, the “Lino Rossi” Research Center of Milan University, Italy, has examined over 200 cases of unexplained fetal and infant death, as it is the national reference center for the application and enforcement of the Italian Law 31/2006 “Regulations for diagnostic post mortem investigation in victims of sudden infant death syndrome (SIDS) and unexpected fetal death” [1]. This law decrees that all infants suspected of SIDS who died suddenly in Italian regions within the first year of age as well as all fetuses who died after the 25th week of gestation without any apparent cause must undergo diagnostic post mortem investigations performed according to specific guidelines. The application of this protocol, which foresees an in-depth anatomo-pathological examination of the autonomic nervous system, including immunohistochemical techniques to highlight the expression of various functional markers, allowed for carrying out a wide range of research studies, many of which have highlighted the harmful effects of prenatal nicotine absorption on the autonomic nervous system in perinatal life. In particular, a significantly increased incidence of hypodevelopment of nuclei prevalently located in the brainstem has been observed in fetuses and infants with smoking mothers compared to victims with nonsmoker mothers [2,3,4,5,6,7].

The aim of this study is to provide evidence of the primary mechanism underlying the perturbed development of brainstem substructures associated with nicotine absorption, especially focusing on neuronal nicotinic acetylcholine (Ach) receptors (nAChRs). These receptors, widely expressed in the fetal nervous system, mainly in neuronal structures that are undergoing major phases of differentiation and synaptogenesis, are responsible for the regulation of many vital phases of brain maturation, by modulating the excitatory neurotransmission of the Ach [8,9,10,11].

This study in particular aims to evaluate if nicotine, one of the many neurotoxic chemicals contained in tobacco smoke, mimicking the effects of the endogenous neurotransmitter ACh, may have affected the nAChR expression in specific brain regions during the most critical periods of development.

## 2. Materials and Methods

Fifty-four brains were collected from 32 sudden fetal death cases (20 females and 12 males, 28–40 gestational weeks) and 22 newborns (10 females and 12 males who died in the first two months of postnatal life), for whom the traditional autopsy procedure, including the examination of the placenta in the case of fetuses, did not reveal a specific cause of death. The acronym “SIUD” (Sudden Intrauterine Unexplained Death) is used for fetal deaths that occur after 28 weeks of pregnancy, and “e-SIDS” (early-Sudden Infant Death Syndrome) is used for newborns who die in the first weeks of life. This was a selected set of cases of perinatal death sent to our Research Center in conformity with the directives of the above-mentioned Italian law n. 31/2006.

A complete medical history, with particular focus on maternal lifestyle habits, was provided for each case. None of the mothers of the 54 victims had any significant pathology. The mothers were asked if they smoked cigarettes. Thirty mothers (56%) reported to have been active smokers before and during pregnancy, while 24 (44%) declared that they had never smoked. As the retrospective assessment of maternal smoking habits may be complicated following a child’s death due to a deep sense of guilt [12], deceptive self-reports were verified by testing the hair of the victims for cotinine, the main metabolite of nicotine characterized by a long half-life and great stability [13]. Four out of the 24 mothers who denied smoking tested positive for cotinine therefore the total number of cases with ascertained nicotine absorption in pregnancy was 34 (63%) (23 cases of SIUD and 11 e-SIDS) thus reducing the number of non-smoking mothers to 20 (37%) (10 SIUD and 10 e-SIDS).

Eight age-matched stillbirths (5 females and 3 males) and 6 newborns (2 females and 4 males) were used as controls for whom a precise cause of death was formulated at autopsy. Specific diagnoses among the fetal deaths included: severe chorioamnionitis (detected in 5 cases) and congenital heart disease (detected in 3 cases). The related diagnoses of neonatal death included 3 cases of congenital heart disease, 1 case of pulmonary dysplasia, 1 case of bronchopneumonia and 1 case of myocarditis. Four of the 14 mothers belonging to the control group had smoked during pregnancy. Table 1 summarizes the features of the cases included in the study.

### 2.1. Ethics Statement

Permission from the Ethics Committee was not required for this study as the Lino Rossi Research Center at Milan University is the national referral center for neuropathological studies under the Italian Law n. 31/2006. This study was part of a legal obligation and therefore the consent of the subjects involved was not required.

### 2.2. Neuropathological Protocol

The neuropathological study was focused on the brainstem, where the main structures controlling the vital functions are located.

**Figure 1:**
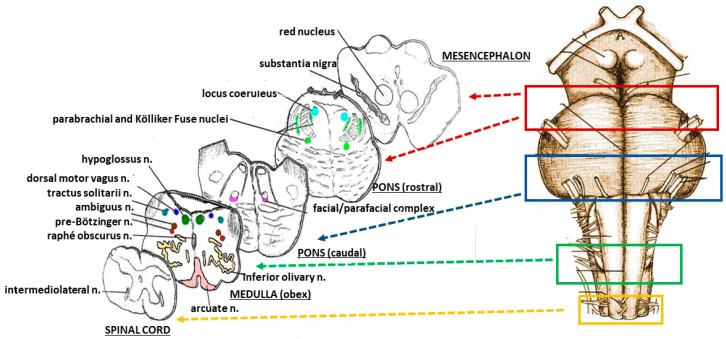
On the right, schematic representation of the sampling from the brainstem. On the left, the histological sections obtained from the specimens, with the indication of the main nuclei and structures to be examined.

Figure 1 shows the brainstem examination methods. On the right, the sampling of three specimens is shown. The first rostral specimen includes the upper third of the pons and the adjacent caudal portion of midbrain; the second specimen is taken from the caudal portion of the pons; the third specimen is from the medulla oblongata around the obex. A last caudal specimen is taken at the border between the medulla oblongata and the spinal cord.

Transverse serial sections of each specimen were taken at 60 µm intervals. Eight to ten 5-µm-thick sections were obtained for each level, two of which were stained with hematoxylin-eosin and Klüver–Barrera for histological examination. On the left–hand side of Figure 1, the representative histological sections obtained from the above-described specimens are shown and the main nuclei and structures to be examined are indicated due to their frequent involvement in sudden fetal and infant deaths, in terms of delayed development (hypoplasia/agenesis) and/or neurotransmitter perturbations.

The microscopic evaluation was focused mainly on the locus coeruleus and the Kölliker-Fuse nucleus, the median raphe nucleus in the rostral pons/caudal mesencephalon, the magnus raphe nucleus, the superior olivary complex, the retrotrapezoid nucleus and the facial/parafacial complex in the caudal pons; the hypoglossus, the dorsal motor vagus, the tractus solitarius, the ambiguus, the pre-Bötzinger, the inferior olivary, the raphé obscurus/pallidus and the arcuate nuclei in the medulla oblongata. 

### 2.3. Immunohistochemical Protocol

The remaining sections obtained from each brainstem specimen were treated with a specific immunohistochemical technique in order to evaluate nicotinic receptor expression. Since the molecular biology of neuronal nAChRs features a multitude of potential subtypes (various homomeric or heteromeric combinations of twelve different nicotinic receptor subunits, designated by Greek letters, followed by Arabic numerals to distinguish variants: α2–α10 and β2–β4) [14,15], this study focused on the α7-nAChRs, which are those essential for normal brain development [16,17].

#### 2.3.1. nAChR Immunohistochemistry

The immunohistochemical method for evaluating α7-nAChRs was carried out using the rabbit polyclonal antibody (aa 22-71, Abcam Ltd., Cambridge, UK) on selected transverse sections. After dewaxing and rehydration, the sections were immersed and boiled in TRIS-EDTA (2-hydroxymethyl aminomethane hydrochloride-ethylenediaminetetraacetic acid) Buffer for antigen retrieval with a microwave oven, after blocking endogenous peroxidase with 3% hydrogen peroxide treatment. The sections were then incubated with diluted 1:167 primary antibody overnight in a wet chamber. The samples were subsequently washed with PBS buffer and incubated with a biotinylated goat anti-rabbit IgG secondary antibody (PK-6101, Vector Laboratories, Burlingame, CA, USA) and processed using the avidin-biotin-immunoperoxidase technique (VEDH-4000, Vector Laboratories). Finally, each section was counterstained with Mayer’s Hematoxylin and coverslipped.

A set of sections from each brainstem sample was used as negative control: the same procedure was applied for staining the nerve tissues without the primary antibody in order to exclude any antibody labeling due to the secondary antibody. If immunostaining occurred in the negative control tissue, the results were considered invalid.

#### 2.3.2. nAChR Immunohistochemistry Quantification

For each selected brainstem nucleus and/or structure, the degree of immunoreactivity was calculated as the number of dark-brown neuronal cells divided by the total number of neurons and expressed as percentage (nAChR immunopositivity index: nAChR-I). nAChR-I was classified as: “Class 0” for no or light staining (negativity); “Class 1” when the index was <10% (weak positivity); “Class 2” with a percentage of immunopositive cells ranging between 10 and 40% (moderate positivity); “Class 3” with an index of >40% of the counted cells (strong positivity).

### 2.4. Statistical Analysis

The results were tabulated and analyzed for differences by comparing pairs of groups with analysis of variance (ANOVA). Statistical calculations were carried out with SPSS statistical software (version 11.5; SPSS Inc., Chicago, IL, USA). The threshold level set for statistical significance was *p* < 0.05.

## 3. Results

### 3.1. Neuropathological Examination

**Figure 2:**
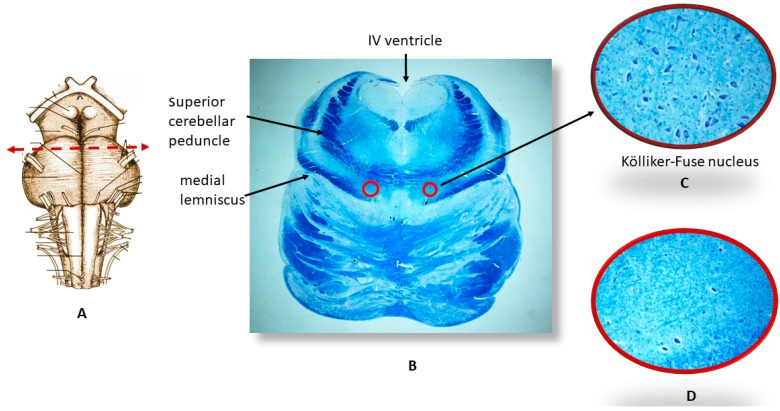
An image series related to the Kölliker-Fuse nucleus (KFN). (**A**) Ventral brainstem image indicating the best level for optimizing the sampling of the KFN; (**B**) Histological section cut at the previously-mentioned level. The circles show the bilateral localization of the KFN, represented at higher magnification in (**C**); At this magnification, a considerable number of large neurons can be seen which are intermixed with smaller cells (interneurons and astrocytes); (**D**) Hypoplasia of the KFN observed in a newborn who died 24 h after birth. (**B**) Klüver Barrera staining, magnification: 0.5×; (**C**,**D**) Klüver–Barrera staining, magnification: 20×.

The histological examination of the brainstems of the 32 SIUD cases and 22 e-SIDS cases showed the hypodevelopment of various nuclei and/or structures checking the vital functions. More precisely, hypoplasia/agenesis of one or more nuclei of the raphé system, of the facial/parafacial complex, the Kölliker-Fuse, the pre-Bötzinger and the arcuate nuclei were observed in 60% of the cases (19 SIUD and 13 e-SIDS, 11 of which died within the first month of life). Combinations of these alterations were frequently found in the same subject. The hypoplasia of the Kölliker-Fuse nucleus (reduced size and number of neurons) was the most frequent finding especially in e-SIDS victims. This alteration was in fact observed in seven fetuses (22% of SIUD cases) and nine newborns (41% of e-SIDS) (Figure 2). No significant brainstem morphological alteration was found in the controls, except for a slight hypoplasia of the arcuate nucleus observed in two cases.

### 3.2. Immunohistochemical Expression of α7 Receptors 

**Figure 3:**
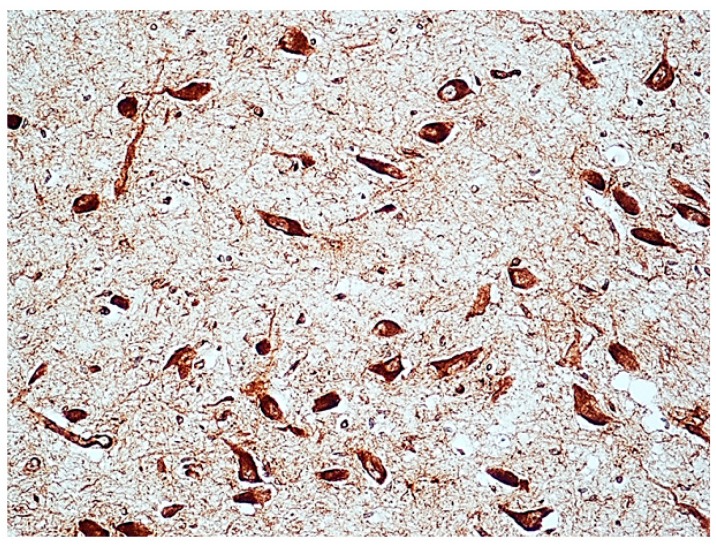
Strong α7-nAChR immunopositivity (“Class 3” of α7-nAChR-Index) in the pontine facial/parafacial complex with normal cytoarchitecture in an SIUD case (38 gestational weeks). α7-nAChR immunostaining; magnification: 40×.

**Figure 4:**
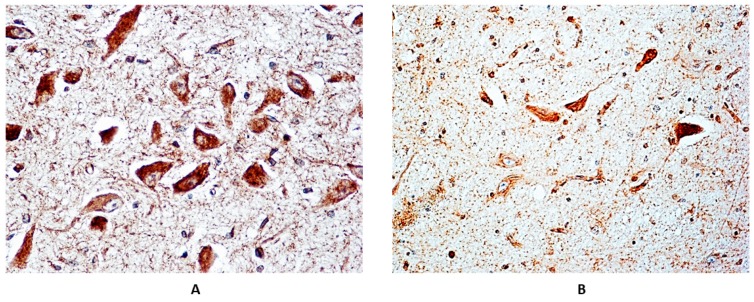
Strong α7-nAChR immunopositivity (“Class 3” of α7-nAChR-Index) in the Kölliker-Fuse nucleus (KFN). (**A**) Normal structure and (**B**) hypoplasia of the KFN in two e-SIDS cases (both died in the first postnatal week). (**A**,**B**) α7-nAChR immunostaining; magnification: 100×.

Dark α7-nAChR membrane-bound intracellular immunoreactivity, indicative of the presence of the receptor protein, was observed in the neurons of brainstem structures with both normal and delayed maturation, in a total of 23 SIUD, 10 e-SIDS, and three controls. More precisely, a percentage of immunostained neurons ranging from 28% to 40% (corresponding to “Class 2” of nAChR-I) was detected in nine SIUD, three e-SIDS and three controls. The percentage of immunostained neurons in the remaining 14 SIUD and seven e-SIDS cases was very high, ranging between 82% and 100% (“Class 3” nAChR-I). Figure 3 shows an example of “Class 3” α7-nAChR-I in the facial/parafacial complex, a pontine structure characterized by the presence of many neurons. In Figure 4, two examples of α7 immunoreactivity of neurons of the pontine Kölliker-Fuse nucleus are shown in cases with normal cytoarchitecture and with hypoplasia, respectively.

### 3.3. Correlations between Immunoexpression of α7-nAChR and Maternal Smoking

A highly significant correlation was observed between α7-nAChR immunopositivity and cigarette smoke absorption in intrauterine life. In fact, all of the 23 SIUD cases and 10 of the 11 e-SIDS cases with smoking mothers showed dark immunostained neurons in many of the main brainstem nuclei. Contrastingly, the mothers were proven non-smokers in all of the cases with α7-receptor immunonegativity *(p* < 0.01). Similarly, three of the mothers of the victims of the control group with α7 subunit hyperexpression were smokers.

Table 2 shows the correlation of the morphological and immunohistochemical alterations with maternal smoking.

## 4. Discussion

Tobacco exposure is known to exert adverse health effects on the health of adults as well as fetuses and newborns [18,19,20]. Smoking during pregnancy causes low birth weight, premature delivery, neonatal morbidity and also mortality [21,22,23,24]. In particular, nicotine, the main pathogenic component of tobacco smoke, hinders foetal brain development when absorbed in intrauterine life, which is a particularly vulnerable phase to toxic agents [7,25,26].

In cases of maternal smoking in pregnancy, carbon monoxide, a gaseous combustion product of cigarette smoke, may readily cross the placenta and bind to fetal hemoglobin [27,28]. The consequent carboxyhemoglobin is not able to release oxygen, thus inducing alterations in the physiological development of fetal organs and tissues, especially those that are particularly susceptible to hypoxic damage, including the brain. Moreover, nicotine is one of the few lipid-soluble substances that can pass through the blood–brain barrier by passive diffusion, thus directly affecting gene expression and the activity of the transmitters essential for the development of the nervous system, such as the acetylcholine (ACh). ACh, the endogenous cholinergic neurotransmitter, plays a fundamental trophic role during various stages of the fetal brain development through synaptic mechanisms mediated by specific receptors, the nicotinic acetylcholine receptors (nAChRs) that are located on the neuronal surface [8,9,10,11]. 

A single nAChR is composed of five subunits around an axis of pseudosymmetry with a small ionic pore. Permeable to cations, this channel usually opens to bind to the endogenous agonist Ach when the action of this neurotrasmitter is necessary for the development of the nervous system, but closes under resting condition. Although it is beyond the scope of this article to discuss the structure of the nAChRs in detail, I would like to underline that these neuronal receptors exist in twelve different subunits (α2–α10 and β2–β4) combined into two different interconvertible conformational states: homomeric receptors, formed by α subunits and heteromeric receptors, composed of combinations of two or more α and β subtypes in the same receptor complex [14,15]. In neurons, the *α*7 nAChR, a homopentamer protein composed of five individual *α*7-subunits plays a specific role in neuronal differentiation, axogenesis and synapse formation [16,17].

Moreover, it is important to note that, compared to other nAChR subtypes, the α7 subunit is characterized by faster kinetics. This raises the possibility that, during key critical developmental periods, the α7-nAChRs could be the primary potential target for neurotoxicants like nicotine, thus causing significant alterations in the normal ACh synaptic turnover rate.

In this study, performed on a wide set of fetuses who died in the last months of pregnancy and newborns suddenly died in the first weeks of postnatal life, with both smoking and non-smoking mothers, a very high correlation was observed between α7 nAChR overexpression and nicotine absorption. Since the main brainstem structures controlling vital functions almost reach maturity in perinatal age, and therefore the activation of nAChRs by ACh is very limited or no longer required, the intense α7 subtype immunoreactivity generally observed in victims with smoking mothers can be attributed to nicotine absorbed during pregnancy. The fact that only these victims show immunopositivity for these receptors, while the same receptors in victims with nonsmoking mothers are immunonegative, clearly indicates the deleterious effect triggered by nicotine. This implies that exogenous nicotine acts as an antagonist of the endogenous ACh by binding inappropriately to nAChRs. Consequently, nicotine alters the regular cholinergic activity due to the inappropriate timing or stimulation intensity negatively affecting the normal function of the brain structures essential for life. 

This mechanism may cause the structural abnormalities that trigger sudden deaths, and in particular the hypoplasia of brainstem nuclei and/or neuronal complexes essential for life reported with high frequency (approximately 60% of cases) in this study. In fact, several studies have reported that stimulation of nAChRs by nicotine causes neuronal inhibition of DNA synthesis, mitotic abnormalities and apoptosis [29,30], leading to a severe decrease in the number of neurons, which is the main feature of hypoplasia.

It is important to note the high frequency of the Kölliker-Fuse nucleus hypoplasia, characterized by the presence of a few, prevalently α7-nAChR immunopositive neurons, prevalently observed in e-SIDS victims with smoking mothers. The Kölliker-Fuse nucleus is a specific brainstem center involved in breathing control. More precisely, experimental studies have demonstrated that the Kölliker-Fuse nucleus, through excitatory synaptic inputs to medullary respiratory regions [31,32], modulates the transition from the inspiration to expiration phase and the dynamic control of the upper airway patency, particularly during expiratory airflow [33,34,35]. Furthermore, the KFN controls the post-apnea airway reflexes essential for postnatal survival. 

## 5. Conclusions

The results of this study show that sudden perinatal death is almost always associated with the activation of nAChRs by nicotine exposure in the womb. These receptors are particularly sensitive to environmental stimuli, especially to exogenous nicotine. In many cases, this induces permanent alterations in cell function and structural impairments of the nerve centers that are essential for life.

We can therefore conclude that aberrant exposure of fetal and neonatal brains to nicotine, through maternal smoking, has detrimental effects on the cholinergic modulation of brain development. This mechanism is the basis of structural changes frequently observed in cases of sudden perinatal death of infants born to mothers who smoked during pregnancy.

Firstly, all pregnant women should be advised that smoking puts their unborn babies at risk of possible structural and functional alterations of vital brainstem centers and to sudden apparently inexplicable death. Moreover, the ineffectiveness of smoking cessation interventions among pregnant women suggests that these interventions should focus on preventing cigarette smoking among teenaged girls. In fact, most women begin smoking as teenagers and then find it difficult to stop smoking in pregnancy due to nicotine addiction.

## Figures and Tables

**Table 1:** Case profiles of the study.

Victims	Sex		Age Range	Death Diagnosis	Maternal Smoking Habit	
	F	M			Smokers	Nonsmokers
**Fetuses**	20	12	28–40 gw	SIUD (*n* = 32)	23	10
	5	3		Control cases (*n* = 8):	2	6
				severe chorioamnionitis (*n* = 5)		
				congenital heart disease (*n* = 3)		
**Newborns**	10	12	0–2 pm	e-SIDS (*n* = 22)	11	10
	2	4		Control cases (*n* = 6):	2	4
				congenital heart disease (*n* = 3)		
				broncopneumonia (*n* = 1)		
				pulmonary dysplasia (*n* = 1)		
				myocarditis (*n* = 1)		

F = female; gw = gestational weeks; M = male; pm = postnatal months; SIUD = sudden intrauterine unexplained death; e-SIDS = early sudden infant death syndrome.

**Table 2:** Distribution of the brainstem morphological and α7-nAChR alterations in SIUD, e-SIDS and control cases, related to maternal smoking.

Brainstem Nuclei with Hypoplasia/Agenesis *	SIUD *n* = 32[23]	Control Fetal Death *n* = 8[2]	e-SIDS *n* = 22[11]	Control Neonatal Death *n* = 6[2]
Kölliker nucleus	7[7]	0[0]	9[9]	0[0]
Raphé nuclei	5[5]	0[0]	4[2]	0[0]
Pre-Bötzinger	5[2]	0[0]	4[4]	0[0]
Facial/parafacial complex	5[5]	0[0]	4[1]	0[0]
Arcuate nucleus	5[5]	1[1]	5[3]	1[0]
α7-nAChR immunopositivity **				
“Class 2” Index	9[9]	2[2]	3[2]	1[1]
“Class 3” Index	14[14]	0[0]	8[8]	0[0]

In brackets, the number of cases with smoking mothers. * A single subject may have more alterations of the nuclei here indicated; ** see “nAChR immunohistochemistry quantification” in Materials and Methods for the explanation of the receptor expression grading; -Statistically-significant differences between hypodevelopment of brainstem nuclei in SIUD and e-SIDS cases with smoker mothers vs. SIUD and e-SIDS with non-smoker mothers: *p* < 0.05; -Statistically-significant differences between α7-nAChR expression in SIUD and e-SIDS cases with smoker mothers vs. SIUD and e-SIDS with non-smoker mothers: *p* < 0.01; nAChR = nicotinic acetylcholine receptor; SIUD = sudden intrauterine unexplained death; e-SIDS = early sudden infant death syndrome.

## References

[1] Constitution of the Italian Republic Law n 31. Regulations for Diagnostic Post-Mortem Investigation in Victims of Sudden Infant Death Syndrome (SIDS) and Unexpected Fetal Death. http://users.unimi.it/centrolinorossi/files/gazz_ufficiale.pdf.

[2] Lavezzi A.M., Ottaviani G., Mauri M., Matturri L. (2007). Biopathology of the dentate-olivary complex in sudden unexplained perinatal death and sudden infant death syndrome related to maternal cigarette smoking. Neurol. Res..

[3] Lavezzi A.M., Matturri L., Del Corno G., Johanson C.E. (2013). Vulnerability of fourth ventricle choroid plexus in sudden unexplained fetal and infant death syndromes related to smoking mothers. Int. J. Dev. Neurosci..

[4] Lavezzi A.M., Corna M.F., Matturri L. (2010). Ependymal alterations in sudden intrauterine unexplained death and sudden infant death syndrome: Possible primary consequence of prenatal exposure to cigarette smoking. Neural. Dev..

[5] Lavezzi A.M., Corna M.F., Repetti M.L., Matturri L. (2013). Cerebellar Purkinje cell vulnerability to prenatal nicotine exposure in sudden unexplained perinatal death. Folia Neuropathol..

[6] Lavezzi A.M., Mecchia D., Matturri L. (2012). Neuropathology of the area postrema in sudden intrauterine and infant death syndromes related to tobacco smoke exposure. Auton. Neurosci..

[7] Lavezzi A.M., Ottaviani G., Matturri L. (2005). Adverse effects of prenatal tobacco smoke exposure on biological parameters of the developing brainstem. Neurobiol. Dis..

[8] Picciotto M.R., Higley M.J., Mineur Y.S. (2012). Acetylcholine as a neuromodulator: Cholinergic signaling shapes nervous system function and behavior. Neuron.

[9] Boyd R.T. (1997). The molecular biology of neuronal nicotinic acetylcholine receptors. Crit. Rev. Toxicol..

[10] Lindstrom J., Anand R., Gerzanich V., Peng X., Wang F., Wells G. (1996). Structure and function of neuronal nicotinic acetylcholine receptors. Prog. Brain Res..

[11] Albuquerque E.X., Pereira E.F., Alkondon M., Rogers S.W. (2009). Mammalian nicotinic acetylcholine receptors: From structure to function. Physiol. Rev..

[12] Shipton D., Tappin D.M., Vadiveloo T., Crossley J.A., Aitken D.A., Chalmers J. (2009). Reliability of self-reported smoking status by pregnant women for estimating smoking prevalence: A retrospective, cross sectional study. Br. Med. J..

[13] Tzatzarakis M.N., Vardavas C.I., Terzi I., Kavalakis M., Kokkinakis M., Liesivuori J., Tsatsakis A.M. (2012). Hair nicotine/cotinine concentrations as a method of monitoring exposure to tobacco smoke among infants and adults. Hum. Exp. Toxicol..

[14] Le Novere N., Corringer P.J., Changeux J.P. (2002). The diversity of subunit composition in nAChRs: Evolutionary origins, physiologic and pharmacologic consequences. J. Neurobiol..

[15] Lindstrom J., Clementi F., Fornasari D., Gotti C. (2000). The structures of neuronal nicotinic receptors. Neuronal Nicotinic Acetylcholine Receptors.

[16] Broide R.S., Lesli F.M. (1999). The alpha7 nicotinic acetylcoline receptor in neuronal plasticity. Mol. Neurobiol..

[17] Falk L., Nordberg A., Seiger Å., Kjaeldgaard A., Hellström-Lindahl E. (2002). The alpha7 nicotinic receptors in human fetal brain and spinal cord. J. Neurochem..

[18] Mathers M., Toumbourou J.W., Catalano R.F., Williams J., Patton G.C. (2006). Consequences of youth tobacco use: A review of prospective behavioural studies. Addiction.

[19] Cornelius M.D., Day N.L. (2009). Developmental consequences of prenatal tobacco exposure. Curr. Opin. Neurol..

[20] Rogers J.M. (2008). Tobacco and pregnancy: Overview of exposures and effects. Birth Defects Res. C.

[21] Wisborg K., Henriksen T.B., Hedegaard M., Secher N.J. (1996). Smoking during pregnancy and preterm birth. Br. J. Obstet. Gynaecol..

[22] Andres R., Day M. (2000). Perinatal complications associated with maternal tobacco use. Semin. Neonatol..

[23] Butler N.R., Goldstein H., Ross E.M. (1972). Cigarette smoking in pregnancy: Its influence on birth weight and perinatal mortality. Br. Med. J..

[24] Dietz P.M., England L.J., Shapiro-Mendoza C.K., Tong V.T., Farr S.L., Callaghan W.M. (2010). Infant morbidity and mortality attributable to prenatal smoking in the U.S. Am. J. Prev. Med..

[25] Lichtensteiger W., Ribary U., Schlumpf M., Odermatt B., Widmer H.R. (1988). Prenatal adverse effects of nicotine on the developing brain. Prog. Brain Res..

[26] Gressens P., Laudenbach V., Marret S. (2003). Mechanisms of action of tobacco smoke on the developing brain. J. Gynecol. Obstet. Biol. Reprod. (Paris).

[27] Soothill P.W., Morafa W., Ayida G.A., Rodeck C.H. (1996). Maternal smoking and fetal carboxyhaemoglobin and blood gas levels. Br. J. Obstet. Gynaecol..

[28] Levin E.D., Slotkin T.A., Slikker W., Chang W. (1998). Developmental neurotoxicity of nicotine. Handbook of Developmental Neurotoxicology.

[29] Atluri P., Fleck M.W., Shen Q., Mah S.J., Stadfelt D., Barnes W., Goderie S.K., Temple S., Schneider A.S. (2001). Functional nicotinic acetylcholine receptor expression in stem and progenitor cells of the early embryonic mouse cerebral cortex. Dev. Biol..

[30] Roy T., Andrews J.E., Seidler F.J., Slotkin T.A. (1998). Nicotine evokes cell death in embryonic rat brain during neurulation. J. Pharmacol. Exp. Ther..

[31] Ezure K., Tanaka I. (2006). Distribution and medullary projection of respiratory neurons in the dorsolateral pons of the rat. Neuroscience.

[32] Segers L.S., Shannon R., Lindsey B.G. (1985). Interactions between rostral pontine and ventral medullary respiratory neurons. J. Neurophysiol..

[33] Dutschmann M., Herbert H. (2006). The Kölliker-Fuse nucleus gates the postinspiratory phase of the respiratory cycle to control inspiratory off-switch and upper airway resistance in rat. Eur. J. Neurosci..

[34] Dutschmann M., Dick T.E. (2012). Pontine mechanisms of respiratory control. Compr. Physiol..

[35] Mörschel M., Dutschmann M. (2009). Pontine respiratory activity involved in inspiratory/expiratory phase transition. Philos. Trans. R. Soc. Lond. B Biol. Sci..

